# Optimisation of Using Low-Grade Kaolinitic Clays in Limestone Calcined Clay Cement Production (LC3)

**DOI:** 10.3390/ma18020285

**Published:** 2025-01-10

**Authors:** Paola Vargas, María Victoria Borrachero, Jordi Payá, Ana Macián, Jorge Iván Tobón, Fernando Martirena, Lourdes Soriano

**Affiliations:** 1 Instituto Universitario de Investigación de Ciencia y Tecnología del Hormigón (ICITECH), Universitat Politècnica de València, 46022 Valencia, Spain; pvarsam@doctor.upv.es (P.V.); vborrachero@cst.upv.es (M.V.B.); jjpaya@cst.upv.es (J.P.); anmabe5@upvnet.upv.es (A.M.); 2 Grupo del Cemento y Materiales de Construcción CEMATCO, Departamento de Materiales y Minerales, Universidad Nacional de Colombia, Medellín 50034, Colombia; jitobon@unal.edu.co; 3 Centro de Investigación y Desarrollo de las Estructuras y los Materiales (CIDEM), Universidad Central Marta Abreu, Villa Clara 50100, Cuba; fmartirena@ecosur.org

**Keywords:** limestone calcined clay cement (LC3), low-grade kaolinite clay, kinetics, mechanical properties, carboaluminates

## Abstract

LC3 (limestone calcined clay cement) is poised to become the construction industry’s future as a so-called low-carbon-footprint cement. Research into this subject has determined the minimum kaolinite content in calcined clays to guarantee good mechanical performance. This study examines the use of clay from the Valencian Community (Spain), which has a lower kaolinite content than the recommended amount (around 30%) for use in LC3 and how its performance can be enhanced by replacing part of that clay with metakaolin. This study begins with a physico-chemical characterisation of the starting materials. This is followed by a microstructural analysis of cement pastes, which includes isothermal calorimetry, thermogravimetry, and X-ray diffraction tests at different curing ages. Finally, this study analyses the mechanical performance of standard mortars under compression to observe the evolution of the control mortars and the mortars with calcined clay and metakaolin over time. The results show that the LC3 mortars exhibited higher compressive strength in the mixtures with higher calcined kaolinite contents, achieved by adding metakaolin. Adding 6% metakaolin increased the compressive strength after 90 days, while 10% additions surpassed the control mortar’s compressive strength after 28 days. Mortars with 15% metakaolin exceeded the control mortar’s compressive strength after just 7 curing days. The hydration kinetics showed an acceleration of LC3 hydration with metakaolin additions due to the nucleation effect and the formation of monocarboaluminate and hemicarboaluminate (both AFm phases). The results suggest the potential for combining less reactive materials blended with highly reactive materials.

## 1. Introduction

LC3 cements (limestone calcined clay cements) represent innovation in the construction industry because of their low carbon footprint. These cement types typically include 50% clinker, 30% calcined clay, 15% limestone, and 5% gypsum. LC3 has been formulated to lower the clinker/cement ratio and, consequently, to reduce anthropogenic CO_2_ emissions associated with ordinary Portland cement (OPC) production [1,2,3]. Due to the wide availability of clays and their thermal activation within the 600–850 °C range [3,4,5,6], energy savings are achieved because the calcination temperatures are lower than those required for the clinkering process, and no CO_2_ emissions are associated with the decarbonation of raw materials. As a CO_2_ reduction strategy, LC3 accounts for 35% fewer direct emissions than those from OPC in the cement industry [1,3,7].

The mechanisms by which LC3 acquires similar mechanical and durability properties to OPC are mainly due to three reactions: (i) the pozzolanic reaction of the calcined clay with portlandite generated during OPC hydration; (ii) the reaction between portlandite and C_3_A (from OPC) with limestone to form monocarboaluminate (Mc) and hemicarboaluminate (Hc) instead of monosulfoaluminate (Ms), favouring ettringite precipitation (AFt phase); (iii) the reaction of aluminate from the calcined clay with limestone to form more carboaluminate phases (Mc, Hc). All of these reactions, together with the filler effect of limestone, lead to the development of a denser microstructure, giving LC3 excellent mechanical and durability performance [3,8].

The technology to produce LC3 cements is now feasible in regions where these raw materials are available. LC3 is especially beneficial for developing economies in Central and South America, India, and some countries in South Africa and East Africa, such as Tanzania [9,10,11,12,13,14,15,16]. In many African countries, however, where limestone shortages make traditional cement production difficult, LC3 offers a viable alternative by using clay, which is more readily available. LC3 production is often more cost-effective because it requires less capital investment and makes use of locally available resources, which encourages economic growth and job creation in the region by reducing the cost of cement. LC3 can also make construction more affordable by promoting infrastructure development in these regions and supporting the Sustainable Development Goals [17], particularly those related to sustainable cities and communities, climate action, and responsible consumption and production.

Due to the availability of clay and limestone worldwide, industrial production tests have been developed in Cuba and India. Notably, the Siguaney Cement Factory, in 2013, made Cuba the first to produce this type of cement on an industrial scale. With its LC3, the compressive strengths of concretes were above 35 MPa after 28 curing days [18,19].

In 2014, some LC3 cements containing 50% clinker, 30% calcined clay (low- and high-quality clays were tested), 15% crushed limestone, and 5% gypsum were manufactured in India. Various types of full-scale building materials were produced, such as microconcrete roof tiles, solid concrete bricks, hollow concrete blocks, window/door frames, and low-strength paving stones. The LC3 with high-quality clay achieved compressive strengths of around 42 MPa in mortars after 28 curing days, outperforming the control cement, which reached 32 MPa. The LC3 blends with low-quality clay were comparable to those of OPC [10].

The clinker ratio employed and the quantity of kaolinitic clay presented in the original clay before calcination are the most critical parameters that can affect LC3 reactivity. Other parameters, such as the sulphate and alkali content, the calcined clay/limestone ratio, and the grinding process, can affect LC3 optimisation [1]. Kaolinitic clays are common in the Earth’s crust, but high-grade kaolin deposits are rare and have applications in other industries, such as cosmetics and rubber. This is reflected in the limited availability and relatively high prices of metakaolin compared to other supplementary cementitious materials (SCMs), such as fly ash, blast furnace slag, and silica fume. Calcined kaolinitic clays (MK) are excellent SCMs, but removing impurities during MK manufacture requires wet processing, drying, and milling, all of which consume energy, produce waste, and increase costs [20,21]. Clay deposits also naturally contain a variety of clay minerals, such as kaolinite, illite, montmorillonite, and non-clay impurities, like quartz, feldspar, mica, rutile, hematite, cristobalite, and calcite, which affect clay activation in terms of the calcination temperature and the quality of the reactive components of clays. Therefore, the excellent characterisation of clays, like that proposed by Tironi et al. [14], is required before they are used to produce LC3.

Avet and Scrivener [22] studied seven kaolinitic clays of different origins and compositions. The kaolinite content in the original clay varied from 95% to 17%. The authors found that the amount of calcined kaolinite had the strongest impact on strength development for up to 7 hydration days. After this time, the strength gain was less dependent on this factor, and the calcined clays that were derived from the clays with more than 45% kaolinite yielded similar results. They concluded that when used in LC3 mixtures, clays with over 65% kaolinite content produced systems with lower carboaluminate phases and higher C-(A)-S-H products.

An interesting study by Zolfagharnasab et al. [23] examined the impact of kaolinite content. The author investigated how kaolinite content affected binary mixtures (70% cement, 30% calcined clay) and ternary mixtures (70% cement, 20% calcined clay, and 10% limestone). The study concluded that kaolinite content had a stronger impact on binary systems than on ternary systems and that the formation of carboaluminate phases compensated for the role of low-grade calcined clay.

In countries like Spain, clays are consumed to manufacture sanitary porcelain, tableware, glazes, and engobes, white pastes for floor and wall tiles, and chamottes for natural stoneware. All this results in only deposits with low kaolinite content remaining, which are not the most recommended ones for use in LC3. The Valencian Community in east Spain is home to the largest terracotta tiles and paving industry, with more than 75% of its production being exported [24,25,26]. The primary deposits are located in Castellón province, and the clay fraction consists of illite and/or kaolinite and/or chlorite and/or interstratified clay minerals. Other mineral phases present are montmorillonite, quartz, gypsum, calcite, and feldspars. As the availability of kaolinitic clays is low, it is challenging for the region to find alternative sources of aluminosilicate minerals and limestone to manufacture LC3.

To improve the low degree of reactivity of kaolinite clays, several studies have been carried out. One is that of Zunino and Scrivener [27], which aimed to find alternatives to improve the reactivity of clays with low kaolinite content (30%). The authors compared two methods: wet sedimentation and air separation. The results showed that kaolinite remained concentrated in fine particles after grinding. With the air classifier method, the kaolinite content increased from 30% to 48%. The process was limited by the degree of clay agglomeration during the grinding process and by the efficiency of the classifier itself. The incorporation of grinding aid-based glycol led to a lower deviation of the air classifier process and increased the kaolinite content to 53%. For the wet sedimentation method, the kaolinite content increased to 60% in the material finer than 0.8 µm and to around 40% in the material finer than 50 µm. The wet sedimentation and air separation methods effectively increased the clays’ reactivity due to the combination of increasing their fineness and their kaolinite content in the fine fraction.

This work aims to contribute to the use of local clays with impurities and low kaolinite content to manufacture LC3 by doping them with metakaolin. The goal is to valorise an abundant natural resource without current applicability due to its composition.

## 2. Experimental Procedures

### 2.1. Materials

The starting materials used for this study were Portland cement type CEM II/A-L 42.5R (OPC), supplied by Cementval S.A. (Puerto de Sagunto, Spain). Commercial-grade metakaolin (MK), supplied by ECC International (Europe), was used as the calcined clay derived from clay with a large quantity of kaolinite. Clay with a small quantity of kaolinite was supplied by KAOSA S.A (Castellón, Spain). Limestone (L) was provided by Cementval S.A. (Puerto Sagunto, Spain). OPC and MK were employed as received, while the KAOSA S.A. clay (C) was milled for 20 min in a Nannetti Speedy ball mill (400 g of C and 98 alumina balls with a diameter of 19 mm) to obtain a similar particle size to cement. This ground clay was calcined at 700 °C for 3 h. The limestone was milled for 15 min (500 g of L and 98 alumina balls with a diameter of 19 mm in a Nannetti Speedy ball mill).

The chemical compositions of all the raw materials were measured by XRF using a Philips Magic Pro Spectrometer, and the results are reported in Table 1. The C was composed mainly of silicon oxide (63.97%) and aluminium oxide (21.81%), which is the second most abundant oxide. The MK contained 52.54% silicon and 41.35% aluminium oxide.

**Figure 1:**
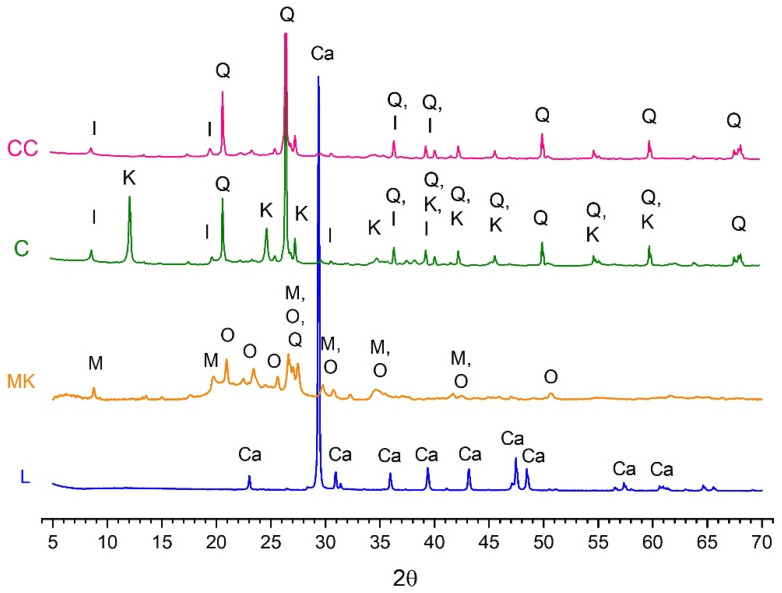
X-ray diffractograms of raw materials. Ca: calcite, I: illite, K: kaolinite, M: muscovite, O: orthoclase, Q: quartz.

The mineralogical composition of raw materials was determined by X-ray diffraction (XRD) using a Bruker AXS D8 Advance diffractometer, with Cu Kα radiation at 20 mA and 40 KV. Diffractograms were recorded for the 2θ range between 5° and 70°, with a steep angle of 0.02° and an accumulation time of 2 s. The diffractograms of the clay (C) and the calcined clay (CC) are presented in Figure 1. Mineral phase identification was performed using Panalytical’s HighScore Plus software (5.1) and its database 2 (Table 2). For the C, kaolinite and illite were identified as the main clay minerals. Small amounts of quartz were also present as companion minerals. After the calcination process, the diffractogram (CC) confirmed the disappearance of the kaolinite peak. In the MK, the identified phases corresponded to muscovite and orthoclase, with quartz as a minor component. The L only contained calcite.

In the C, the kaolinite content was calculated by the thermogravimetric method (TG) based on the mass loss during the dehydroxylation of kaolinite within the 400–700 °C range [28,29]. The test revealed a kaolinite content of 26.87% in the C. The crystalline and amorphous alumina and silica content in the CC and MK were calculated according to [30,31]. In the CC, amorphous silica accounted for 15.2% and amorphous alumina accounted for 11.3%, while in the MK, they accounted for 31% and 21.7%, respectively.

**Figure 2:**
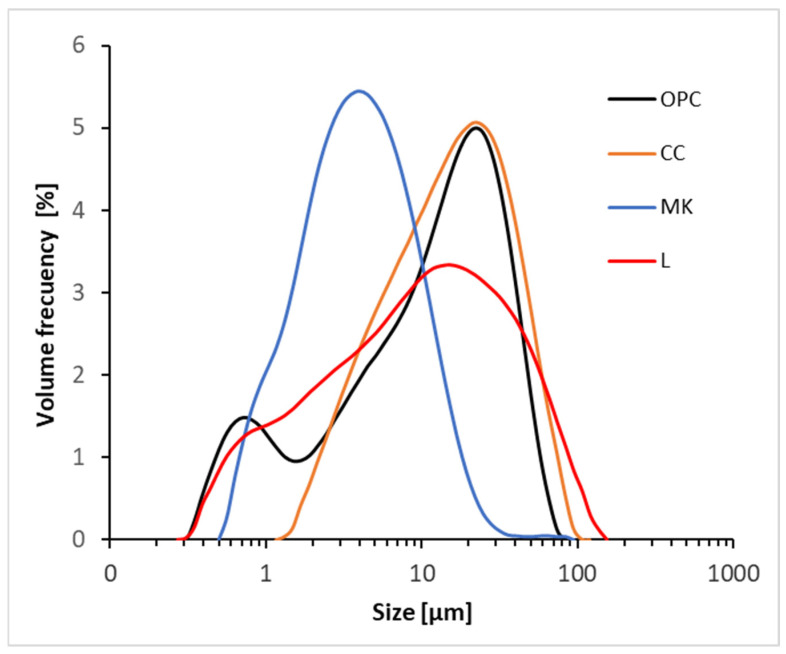
Particle size distribution of raw materials used for preparing LC3 cements.

The particle size distribution of the raw materials was measured by laser diffraction using a Malvern Instruments Mastersizer 2000. OPC measurements were taken using isopropanol or deionised water, applying 1 min of ultrasonic stirring. Figure 2 shows the particle size distribution of all the assessed materials. Table 3 summarises the main granulometric parameters and specific gravity, determined according to UNE 80103 [32]. The grinding process was carried out to obtain a similar particle size distribution to that of OPC. For all the raw materials, the D10 and D50 values were similar to those of OPC. The D90 in the CC (42.31 µm) and L (52.31 µm) were larger than in the OPC (33.83 µm). In the L, the proportion added to the mixtures was very low, as indicated by the blended LC3 proportions shown in Table 4. Therefore, the impact of adding this thicker material was minimal.

### 2.2. Mixture Dose

The systems proposed in this research are based on a typical LC3 composition, where the CC-to-L ratio is 2:1. Different LC3 dry mixes were prepared by mixing materials. As OPC is a type II cement with L addition, it was only necessary to add C and to adjust the L to a percentage of 2.6 wt% to ensure the 2:1 ratio (CC/L). The cement SO_3_ content was 3.3%. Therefore, gypsum was not added. The total sulphate content was calculated according to ASTM C563 (ASTM, 2020). Finally, the OPC, L, MK, and CC mixtures were prepared by placing 470 g of each in a V-shaped solid mixer homogeniser (Comecta model VS-5) for 15 min. In this study, the LC3 control mixture was made with MK and called LC3-MK. MK was selected as a calcined kaolinitic clay with a high grade of kaolinite before the calcination process, which results in a high degree of reactivity. The LC3-CC mixture was made from C, whose kaolinite content was 26.87% before calcination and is considered a low-grade source of calcined kaolinite. Three percentages of the CC and MK mixtures were studied to evaluate the effect of the grade of calcined kaolinite: LC3-6%MK, LC3-10%MK, and LC3-15%MK, with CC/MK proportions of 24/6, 20/10, and 15/15, respectively. Table 4 summarises the doses of the studied mixtures.

### 2.3. Test Methods

Isothermal calorimetry was used to study the hydration kinetics of the LC3 mixtures (TAM Air 8-channel, TA instruments, New Castle, DE, USA). For each sample, 6.40 g of paste was poured into an admix glass ampoule at a w/c ratio of 0.50. The ampoule was hermetically sealed and placed inside the calorimeter at a maintained temperature of 25 ± 0.02 °C. Additionally, 3 g of deionised water was used as a reference. The heat flow and accumulated heat of hydration were measured for 72 h. The hydrates that formed in the LC3 pastes were assessed by a TGA after 7, 28, and 90 curing days. The hydration process of the pastes was stopped by immersing the ground/fractured pastes in acetone, followed by vacuum filtration. The pastes were oven-dried at 65 °C for 30 min to evaporate the acetone. The TGA was performed with a Mettler-Toledo TGA SF/1100 at a heating rate of 10 °C/min from 35 °C to 600 °C in a nitrogen atmosphere (flow rate of 75 mL/min). The samples were placed inside a sealable aluminium crucible with a lid and a micro-pinhole. The crucible was sealed to create a self-generated atmosphere, allowing the dehydration processes to be separated on the TG curve as water vapour was released from the sample [33]. The mineralogical characterisation of the hydrated pastes after 28 curing days was carried out by powder X-ray diffraction (XRD) in a Bruker AXS D8 Advance diffractometer, with Cu Kα radiation at 20 mA and 40 KV. Diffractograms were recorded for the 2θ range between 5° and 70°, with a steep angle of 0.02° and an accumulation time of 2 s. Mineral phase identification was performed with Panalytical’s HighScore Plus software and its database (Table 2). Micrographs were taken to evaluate the pastes’ microstructure by field emission scanning electron microscopy (FESEM–ULTRA 55 Zeiss Oxford instruments) containing an X-ray energy-dispersive detector (EDX, Oxford Instruments, Abingdon, UK). Images were acquired at 2 kV, with a working distance between 6 and 8 mm. EDX analysis was run at 15 kV. Finally, the samples were covered with carbon using high vacuum-coating equipment (BAL-TEC SCD 005).

Cubic mortar specimens (4 × 4 × 4 cm^3^) with standardised sand were prepared at a binder/sand ratio of 1:3 and a water/binder ratio of 0.5 following the procedures described in UNE 196-1 [34]. Compressive strength tests were performed according to European standard UNE 196-1 (AENOR, 2005) after 7, 28, and 90 curing days. After demoulding, the mortars were immersed in a saturated lime solution at 23 °C until reaching the testing age. The test was run on a 3000 kN (accuracy of ±1%) compression IBERTEST MPU-60 testing machine.

## 3. Results

### 3.1. Hydration Kinetics by Isothermal Calorimetry

**Figure 3:**
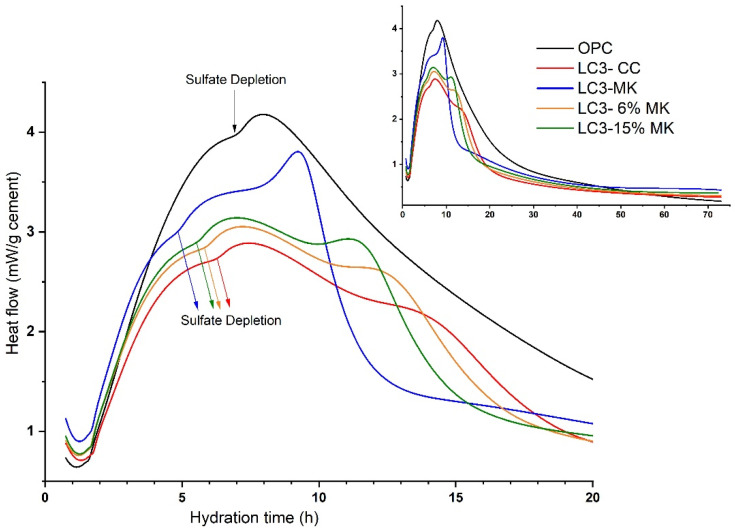
Heat flow rates of the OPC and LC3 systems with different MK additions and 30% CC.

This technique aims to determine the influence of different grades of calcined kaolinite contents on LC3 by combining MK at different percentages. Figure 3 shows the heat flux curves (0–72 h) normalised according to the cement content. For the OPC, the maximum heat flux released was observed to be centred at around 8.3 h. This peak is associated with both C_3_S hydration, with the consequent precipitation of C-(A)-S-H gels, and with renewed C_3_A dissolution, leading to ettringite formation [35,36]. The sulphate depletion peak for a well-balanced cement usually appears after the maximum C_3_S hydration peak [37,38]. OPC is undersulphated for this reason. This finding was confirmed by analysing the heat flow’s first derivative, as Canbek et al. proposed [39]. Sulphate depletion in the OPC occurred at around 6.8 h. Da Silva Andrade et al. [40] concluded that using SCMs with higher alumina content typically led to earlier sulphate depletion and required more gypsum. The increased amount of gypsum in the LC3 mixtures can be attributed to two factors: the nucleation effect and the alumina content. In the present research work, no extra amount of gypsum was added. Thus, if the OPC was already undersulphated, the LC3 mixtures would also be undersulphated. This observation is supported by the curves of the LC3 mixtures shown in Figure 3. Sulphate depletion can be seen at 5.1, 5.5, 5.7, and 6.3 h for the LC3-MK, LC3-15%MK, LC3-6%MK, and LC3-CC mixtures, respectively. This behaviour was observed in our blends, with the LC3-CC mixture’s sulphate depletion occurring at the oldest reaction age due to its lower reactive alumina content and larger mean diameter of particles, in contrast to the mixtures containing MK.

For the LC3 blends, an interesting phenomenon occurred, where three overlapping peaks were observed, with different characteristics depending on the mixture. The first peak was attributed to C_3_S hydration and overlapped two additional peaks. In undersulphated LC3 systems, authors like Canbek et al. [39] have detected, through in situ XRD analysis, the presence of hemicarboaluminate after 16 h. The system’s undersulphation accelerated gypsum depletion and the formation of Afm phases. Therefore, one of these peaks could be due to the formation of carboaluminates.

**Figure 4:**
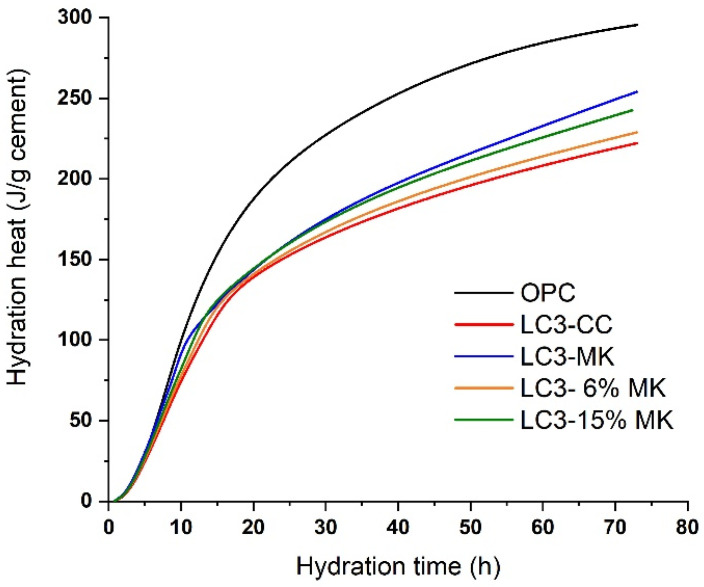
Heat flow rates of the OPC and LC3 systems.

Figure 4 shows the cumulative hydration heat data of both the LC3 and OPC systems, normalised to the total cement content. The hydration heat rose with the addition of MK content. Specifically, for the LC3-CC mix with a lower calcined kaolinite content, the hydration heat was 222 J/g of cement. For the LC3 with 6% MK, it rose to 229 J/g. The hydration heat was 242 J/g for the sample with LC3-15% MK, and it reached 254 J/g for the control-blended LC3-MK. In comparison, the hydration heat for the OPC was 295.4 J/g of cement. Considering the proportion of OPC used in the LC3 mixtures, which was 67.4%, the dilutive effect suggests a reaction heat of approximately 199 J/g of cement. All of the LC3 samples exceeded this value, which indicates the formation of new reaction products (pozzolanic products and the formation of AFm phases) and accelerated cement hydration, likely due to the nucleation effect provided by the supplementary materials included in their formulation.

### 3.2. Thermogravimetry Analysis

**Figure 5:**
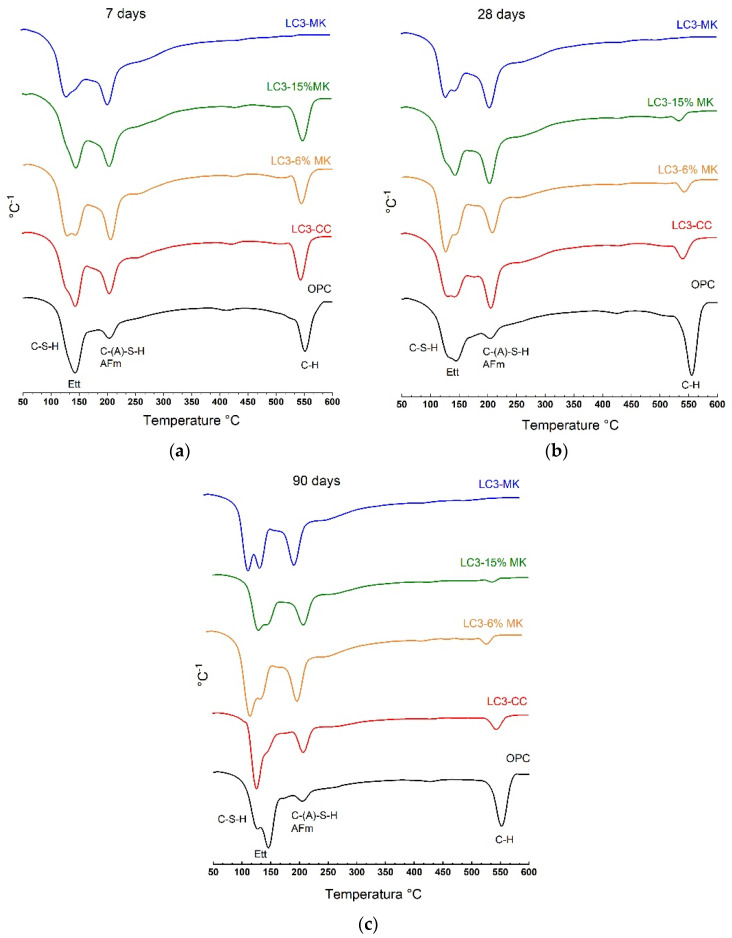
DTG curves (50–600 °C) of the hydrated OPC and LC3 samples after (**a**) 7 days, (**b**) 28 days, and (**c**) 90 days.

The DTG curves of all the samples (OPC and LC3 pastes) tested after 7, 28, and 90 curing days appear in Figure 5. The percentage values of the fixed lime (C-H fixed) are found in Table 5, calculated after considering the percentage of OPC in the samples as proposed by [41]. This table also includes the bond water (BW) percentage values corresponding to the dehydration of the main reaction products formed during the hydration of the OPC and LC3 blends. According to [23], the intervals of decomposition of the combined water in the products that formed during the hydration of the LC3 mixtures, and their typical classification, were as follows: (i) at around 100 °C, water loss is attributed to ettringite (Ett-AFt phase) and C-S-H; (ii) at around 150 °C, it is attributed to monocarboaluminate (Mc) and hemicarboaluminate (Hc) (both AFm phases); (iii) at around 180 °C, it is attributed to monosulphfate (Ms-AFm phase); (iv) at around 180–210 °C, it is attributed to (C-A-H) and (C-(A)-S-H); (v) at around 400–530 °C, it is attributed to portlandite (C-H) dehydroxylation. Typically, the weight range of loss is calculated using an alumina crucible. In this study, we employed sealed aluminium crucibles, which delayed the appearance of mass losses. With this type of sealed crucible, the decomposition peaks of ettringite and C-S-H seemed to overlap at a temperature range of 120–150 °C. C-A-H and C-(A)-S-H decomposed above 200 °C, and portlandite dehydroxylation occurred at 520–600 °C [41].

In this study, according to the condition test, the temperature range for the TG calculations was adopted as 100–150 °C for the dehydration of C-S-H and ettringite, and as 150–300 °C for the dehydration of the AFm (Hc and Mc), C-A-H, and C-(A)-S-H phases, and, finally, at 475–550 °C for C-H dehydroxylation. All LC3 mixtures were studied and are represented in Figure 5. They showed more developed peaks within the temperature interval, attributed to the AFm, C-A-H, and C-(A)-S-H phases, compared to the OPC paste. This observation is corroborated in Table 5. The amount of water within the temperature range of 150–300 °C for all LC3 mixtures was higher at all the curing ages than in the OPC paste. Distinguishing whether more AFm phases or more C-(A)-S-H phases form is a challenge for thermogravimetry. However, other authors, like Avet et al., pointed out that, for clays with a kaolinite content over 65%, the presence of C-(A)-S-H phases predominates over carboaluminates formation [22]. When we observed hydration evolution over time, after 7 days, the mixtures with a higher calcined kaolinite content (LC3-MK) and medium calcined kaolinite content (LC3-15%MK) had already consumed the system’s free C-H. Nevertheless, as the calcined kaolinite content decreased in the LC3-CC and LC3-6%MK mixtures, CH was available until 90 curing days. This finding indicates the low pozzolanic reactivity of the CC obtained from the low kaolinitic clay used in this research.

### 3.3. Hydrated Phases by Powder X-Ray Diffraction

**Figure 6:**
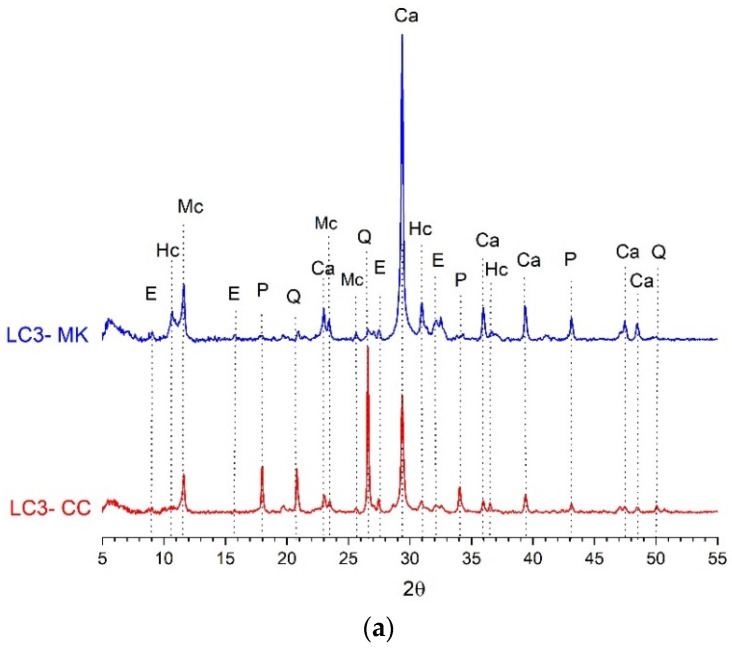

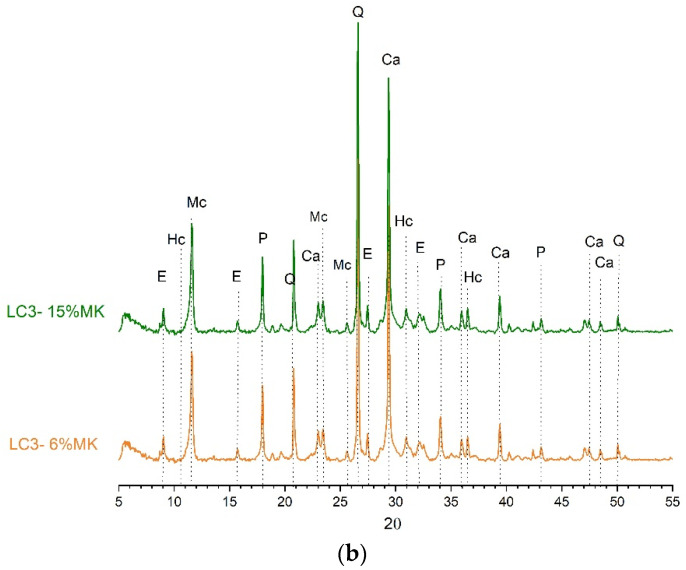
XRD patterns of (**a**) the LC3-CC and LC3-MK pastes cured for 28 days; (**b**) the LC3-6%MK and LC3-15%MK pastes cured for 28 days. Ca: calcite, E: ettringite Hc: hemicarboaluminate; Mc: monocarboaluminate; Q: quartz; P: portlandite.

Figure 6 shows the XRD patterns of the LC3 pastes after 28 curing days. The diffractogram of the LC3-MK control sample reveals the main peaks typical of the hydration process of LC3, including calcite (Ca) from L, hemicarboaluminate (Hc), and monocarboaluminate (Mc)—the principal AFm phases due to the reaction of L with C_3_A (from OPC). Additionally, portlandite (P) was present instead of monosulphoaluminate (Ms), which promotes ettringite precipitation (Aft phase) [22,42]. For all of the LC3 mixtures, the peaks corresponding to ettringite, Mc, and Hc appeared in the diffractogram. The most evident finding was in the main P peak shown in the LC3 mixtures with CC. This peak was significantly higher than that of the LC3-MK, which agrees with the curves of the thermogravimetric results and corroborates the low pozzolanic reactivity of the CC.

### 3.4. Microstructural Analysis of the Hydrated Phases by SEM

**Figure 7:**
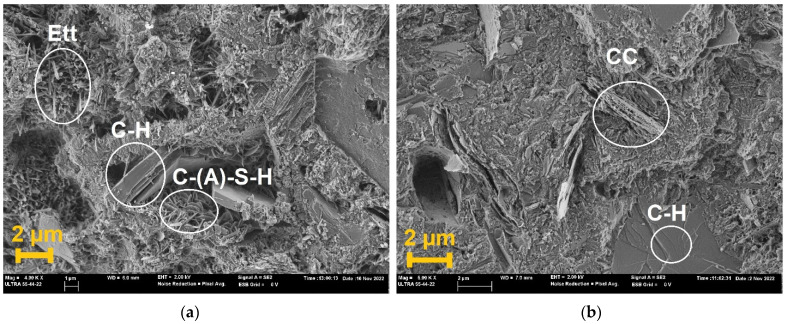

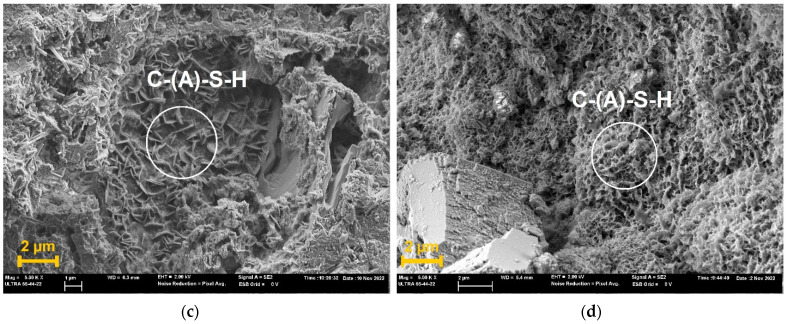
SEM micrographs of (**a**) LC3-CC at 7 days. (**b**) LC3-CC at 28 days. (**c**) LC3-MK at 7 days. (**d**) LC3-MK at 28 days.

Micrographs of the pastes are shown in Figure 7. In this part of the study, only the two extremes of the LC3 mixtures were studied: LC3-CC and LC3-MK. The micrograph of the LC3-CC after 7 curing days (Figure 7a) reveals many needle-shaped crystals corresponding to ettringite, intermixed with C-S-H and honeycombed C-(A)-S-H. In this sample, the presence of portlandite (C-H) crystals and porosity between the hydrated phases was noted. After 28 days, the micrograph of LC3-CC (Figure 7b) showed the presence of unreacted clay and P crystals. This behaviour was derived from the low reactivity of the CC. In contrast, the internal microstructure of the LC3-MK (Figure 7c) sample after 7 days lacked P crystals and demonstrated greater development for the C-S-H and C-S(A)-H phases. After 28 days, the micrograph of LC3-MK (Figure 7d) was characterised by microstructure densification, mainly by C-(A)-S-H formation. These findings confirm its high pozzolanic activity, which can increase matrix density.

### 3.5. Compressive Strength in Mortars

**Figure 8:**
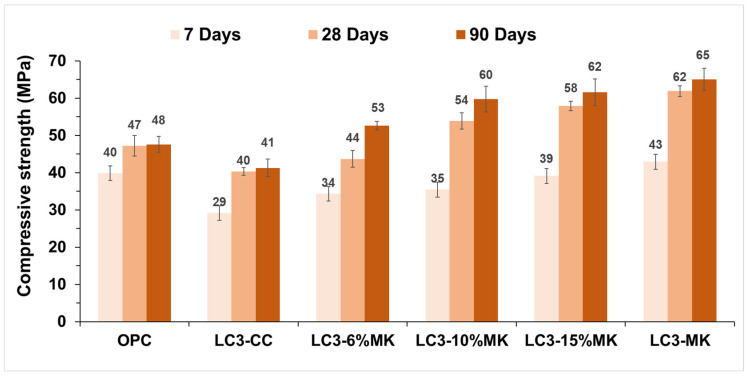
Compressive strength of OPC and LC3 mortars after 7, 28, and 90 curing days.

Figure 8 shows the compressive strength results of the control mortar (100% OPC) and LC3 systems. At all curing ages, the LC3 with CC without MK addition (LC3-CC) performed the worst. Between 28 and 90 days, there was no difference in strength gain. Despite this, it achieved 41 MPa after 90 curing days, which is equivalent to the 85% performance of the OPC. The LC3-MK mortar showed greater mechanical strength than the control mortar after 7 curing days. This result is consistent with findings reported by other researchers. Avet and Scrivener [22] confirmed that the mortar strength after 7 curing days was directly related to the amount of calcined kaolinite present. After 7 days, the same authors [22] noticed that the strength gain of LC3-50 blends with calcined clay, especially those containing more than 45% calcined kaolinite, tended to become less significant.

The mortars with over 40% calcined kaolinite obtained the most significant strength gains between 7 and 28 curing days. The 6% MK addition improved the strength of the LC3 CC mix at all ages and exceeded the OPC strength by 10% after 90 curing days. Interestingly, the mortars with LC3-10% MK, LC3-15% MK, and LC3-MK showed a 19 MPa increase in strength as the curing period prolonged from 7 to 28 days. Similar results have been reported by Scrivener et al. [43], who concluded that clays containing between 40% and 75% calcined kaolinite exhibited the most substantial strength gain during this period. The authors also concluded that when the calcined kaolinite content was as low as 40%, mortars were also able to reach the strength of OPC after 28 days.

The blend containing 15% MK and 15% CC (LC3-15%MK) achieved a compressive strength of 62 MPa after 90 days of curing. In comparison, the blend with 30% MK (LC3-MK) obtained a compressive strength of 65 MPa. This demonstrates a strength increase of 30% to 35% when compared to ordinary Portland cement (OPC). Despite this significant gain, the system at 15% was optimal enough.

## 4. Conclusions

Upon careful analysis of the results reported for the pastes and mortars, the main conclusions are listed as follows:

The hydration heat of the LC3 mixes was lower than for the OPC. The formation of AFm, AFt, and pozzolanic reactions did not compensate for the 50% reduction in cement content. The presence of the C_3_S hydration peak remained consistent regardless of using mixtures with varying calcined kaolinite contents. However, the calcined kaolinite content influenced the sulphate depletion and the subsequent C_3_A reaction. As the percentage of metakaolin (MK) in the pastes increased, the time until sulphate depletion occurred decreased. This is attributed to the higher amount of reactive alumina and the smaller particle size resulting from the increased amount of MK.

The thermogravimetry studies demonstrated that the LC3-MK paste fixed all the portlandite after 7 curing days. The fixed lime percentages decreased as the calcined kaolinite percentages in the mixtures decreased. Regarding the reaction products that formed in the LC3 mixtures, the greater development of the C-(A)-S-H and AFm phases compared to the OPC control was confirmed.

The compressive strength of the LC3-6%MK system was similar to or higher than that of the control mortar (OPC)’s strength after 90 curing days. With 10% MK (LC3-10% MK), it could sufficiently obtain a compressive strength above that of OPC after 28 curing days.

In terms of mechanical performance, the system comprising 15% MK and 15% CC is considered the optimal choice among the combinations of both materials.

In conclusion, enhancing clay with a small amount of kaolinite by adding pure MK improves its compressive strength. Thus, this could become a way to use low-kaolinitic clays. Depending on the specific application, utilising clays with 30% kaolinite content (LC3-CC) or modified mixtures may be adequate to offer superior performance.

## Figures and Tables

**Table 1:** Chemical compositions of the materials used in this study, as determined by XRF (LOI was determined at 950 °C for 1 h).

Oxide Composition [%]	OPC	C	L	MK
SiO_2_	15.66	63.97	-	52.54
Al_2_O_3_	3.65	21.81	0.29	41.35
Fe_2_O_3_	2.49	2.50	-	4.36
CaO	62.25	2.36	54.71	0.07
MgO	1.92	0.48	1.51	0.19
Na_2_O	0.12	0.17	-	0.26
K_2_O	1.03	3.19	0.07	0.64
SO_3_	3.30	-	0.38	-
TiO_2_	0.00	-	0.01	-
P_2_O_5_	0.14	0.03	0.01	-
MnO	0.02	-	-	-
LOI	9.41	5.48	43.01	0.60

**Table 2:** Mineral phase identification.

Key	Mineral Phase	Chemical Formula	Code Xpert
Ca	Calcite	CaCO_3_	00-005-0586
l	Illite	(K,H_3_O)Al_2_Si_3_ AlO_10_ (OH)_2_	00-026-0911
K	Kaolinite	Al_2_Si_2_O_5_(OH)_4_	01-080-0886
M	Muscovite	KAl_2_Si_3_AlO_10_(OH)_2_	00-007-0025
O	Orthoclase	KAlSi_3_O_8_	01-075-1592
Hc	Hemicarboaluminate	Ca_4_Al_2_O_6_(CO_3_)_0.5_(OH)·11.5H_2_O	00-041-0221
Mc	Monocarboaluminate	Ca_4_Al_2_O_6_(CO_3_)·11H_2_O	00-041-0219
P	Portlandite	Ca(OH)_2_	00-044-1481
E	Ettringite	Ca_6_Al_2_(SO_4_)_3_(OH)_12_·26H_2_O	00-041-1451
Q	Quartz	SiO_2_	01-085-0796

**Table 3:** Particle size distribution values and specific gravity of raw materials.

Materials	Acronym	Particle Size Average (μm)	D10 (µm)	D50 (µm)	D90 (µm)	Specific Gravity
CEM II/A-L 42.5 R	OPC	14.89	0.93	11.63	33.83	3.15
Calcined Clay	CC	19.50	3.76	14.80	42.31	2.64
Limestone	L	19.78	1.21	9.60	52.31	2.71
Metakaolin	MK	5.72	1.31	4.08	11.83	2.47

**Table 4:** Formulations of the control and LC3 mixtures to fabricate mortars and pastes.

Acronym Blend	OPC [wt%]	CC [wt%]	MK [wt%]	L [wt%]
OPC	100	-	-	-
LC3 -CC	67.4	30	-	2.6
LC3 -MK	67.4	0	30	2.6
LC3-6% MK	67.4	24	6	2.6
LC3-10% MK	67.4	20	10	2.6
LC3-15% MK	67.4	15	15	2.6

**Table 5:** Percentage values of the fixed lime (C-H fixed) and bound water after 7, 28, and 90 curing days.

Sample	Fixed Lime [%]			Bound Water [%]					
	7 Days	28 Days	90 Days	7 Days	28 Days	90 Days	7 Days	28 Days	90 Days
				100–150 °C			150–300 °C		
OPC				4.0	3.5	5.8	4.3	4.1	4.5
LC3-MK	98.9	97.0	98.0	4.3	4.7	6.3	6.4	7.1	7.7
LC3-15% MK	37.1	84.7	92.1	3.2	4.1	5.0	5.3	6.6	7.1
LC3-6% MK	19.4	67.0	78.8	3.8	5.9	5.4	4.9	6.3	6.9
LC3-CC	12.0	46.2	61.1	3.3	3.4	5.9	4.7	5.7	6.1

## Data Availability

The original contributions presented in the study are included in the article; further inquiries can be directed to the corresponding author.
